# Celebrating 20 years of open access publishing at BMC Musculoskeletal Disorders

**DOI:** 10.1186/s12891-020-03785-2

**Published:** 2020-11-21

**Authors:** Ciarán Martin Fitzpatrick, Amanpreet Athwal

**Affiliations:** BMC Series, Springer Nature, 4 Crinan Street, London, N1 9XW UK

## Abstract

Twenty years ago, on October 23, the first article published by *BMC Musculoskeletal Disorders* appeared free online. Over 5700 publications later, we celebrate our anniversary as the largest Open Access journal in the ‘Orthopaedics and Sports Medicine’ and ‘Rheumatology’ fields. Our ‘open, inclusive, and trusted’ ethos, along with our efficient and robust peer review services, are recognized by the musculoskeletal field.

The early pioneers of *BMC Musculoskeletal Disorders* pushed the Open Access publishing model, in order to better support the needs of both the clinical and research communities. We pride ourselves on the continual innovation of author services, data transparency, and peer review models. These advances would not have been possible without your efforts - so a massive thank you to all the authors, editorial teams, and reviewers who have contributed to our success. Excellent reviewers are the nucleus of any thriving journal, and we have been lucky to collaborate with so many talents.

**Supplementary Information:**

The online version contains supplementary material available at 10.1186/s12891-020-03785-2.

## Main text

Twenty years ago, on October 23, the first article published by *BMC Musculoskeletal Disorders* appeared free online [1]. Over 5700 publications later, we celebrate our anniversary as the largest Open Access journal in the ‘Orthopaedics and Sports Medicine’ and ‘Rheumatology’ fields. Our ‘open, inclusive, and trusted’ ethos, along with our efficient and robust peer review services, are recognized by the musculoskeletal field.

The early pioneers of *BMC Musculoskeletal Disorders* pushed the Open Access publishing model, in order to better support the needs of both the clinical and research communities. We pride ourselves on the continual innovation of author services, data transparency, and peer review models. These advances would not have been possible without your efforts - so a massive thank you to all the authors, editorial teams, and reviewers who have contributed to our success. Excellent reviewers are the nucleus of any thriving journal, and we have been lucky to collaborate with so many talents.

We take this opportunity to look back on some highlights and milestones in our partnership with our diverse community:

- 2,350,000 article downloads in 2011; one meta-analysis on stabilisation exercises for back pain is our most clicked study ever with 123,000 reads [2].
- 13,332 citations in 2019; the disabilities of the arm, shoulder and hand (DASH) outcome questionnaire has been cited an impressive 581 times [3].
- Recent article collections on osteoarthritis, work and musculoskeletal health, and imaging provided a venue for deeper investigation into important topics.

**Fig. 1:**
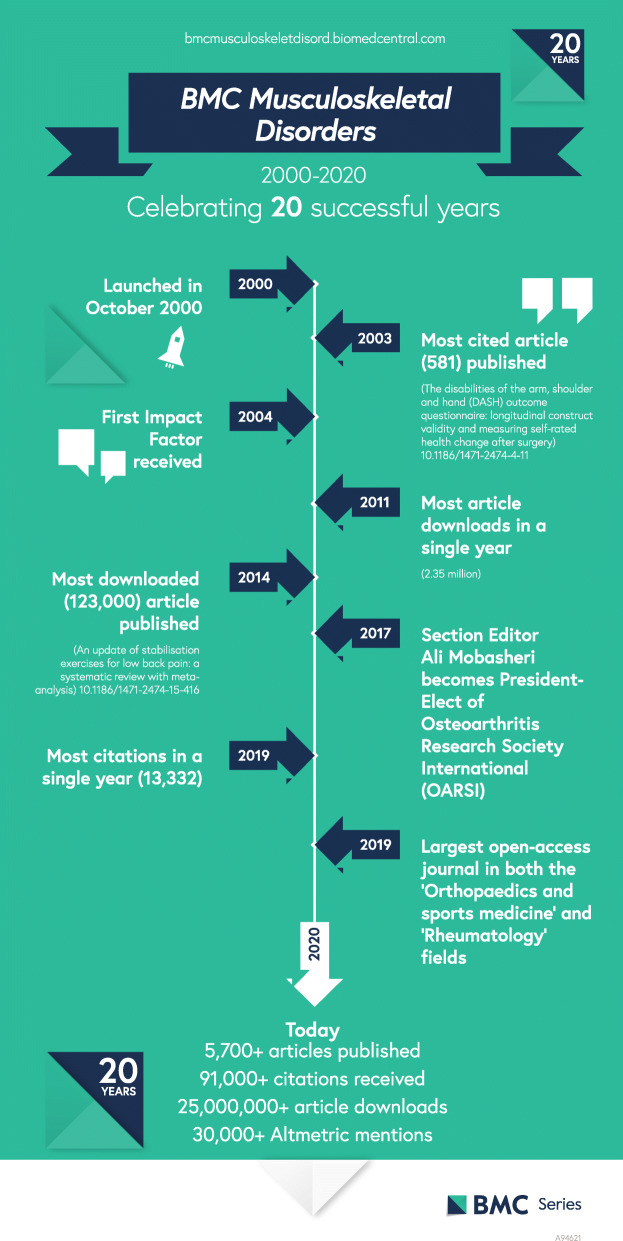
Infographic celebrating 20 successful years of *BMC Musculoskeletal Disorders*

As part of the *BMC Series* policy*,* we do not make editorial decisions on the basis of the interest of a study or its likely impact. Studies must be scientifically valid, for research articles this includes; a scientifically sound research question, the use of suitable methods and analysis, and following community-agreed standards relevant to the research field. Our leadership in editorial policies has allowed thousands of authors to maximise the reach and readership of their work around the world, with 25,000,000+ article downloads and 91,000+ citations to date.

As the field acknowledges that OA is here to stay and continues to thrive, we highlight some future advances in the field that we excite us:

- **Preprint publishing** provides a service for the rapid dissemination of preliminary research findings. A preprint is a finalized draft of a research manuscript that is shared publicly online pre-peer review. *BMC Musculoskeletal Disorders,* in partnership with Research Square*,* launched **In Review** to facilitate this option. Authors benefit by receiving credit, feedback, and visibility for their submitted work. Manuscripts can be tracked, allowing authors to view from when reviewers are invited to when reports are received. 500+ preprints have already been posted on our **In Review** site.
- Rapid progress in **musculoskeletal imaging** and **digital health** is reaching a tipping point, with these applications finally becoming commonly used in clinical practice. **Artificial intelligence** and **data science** fuel this transition to the mainstream, and *BMC Musculoskeletal Disorders* will highlight these topics.
- Empowered **patient and public involvement** in research is something that delights us [4, 5]. The notion that research and clinical practice be geared towards the needs of patients, as well as involving them, is increasingly realized by funding and healthcare organisations. *BMC Musculoskeletal Disorders* is working to recognize and engage patients.

Scientific journals should benefit society, contributing to the patient and research communities we serve. This is *BMC Musculoskeletal Disorders* expectation for the next 20 years, as we take this moment to look into the horizon whilst celebrating with birthday cake.

## Supplementary Information

**Additional file 1.** Supplementary Video. https://www.youtube.com/watch?v=0gq36uhr2jg.

## Data Availability

Not applicable.
