# Supplementary material for: Celebrating 20 years of open access publishing at BMC Musculoskeletal Disorders
Source: BMC Musculoskelet Disord. 2020 Nov 21;21:771. doi: 10.1186/s12891-020-03785-2 (PMC7680582; doi:10.1186/s12891-020-03785-2)
Supplement: Supplementary file 1 — Additional file 1. Supplementary Video. https://www.youtube.com/watch?v=0gq36uhr2jg. [file 12891_2020_3785_MOESM1_ESM.docx]

**Supplementary Video**

<https://www.youtube.com/watch?v=0gq36uhr2jg>
